# Potential Molecular Pathways Related to Pulmonary Artery Aneurysm Development: Lessons to Learn from the Aorta

**DOI:** 10.3390/ijms21072509

**Published:** 2020-04-04

**Authors:** Jorge Nuche, Julián Palomino-Doza, Fernando Arribas Ynsaurriaga, Juan F. Delgado, Borja Ibáñez, Eduardo Oliver, Pilar Escribano Subías

**Affiliations:** 1 Centro de Investigaciones Biomédicas en Red de enfermedades CardioVasculares (CIBERCV), 28029 Madrid, Spain; jorge-nuche@hotmail.com (J.N.); julian.palomino@salud.madrid.org (J.P.-D.); fernando.arribas@salud.madrid.org (F.A.Y.); juan.delgado@salud.madrid.org (J.F.D.); bibanez@cnic.es (B.I.); 2 Servicio de Cardiología, Hospital Universitario 12 de Octubre, Instituto de Investigación Sanitaria Hospital 12 de Octubre (imas12), 28041 Madrid, Spain; 3 Centro Nacional de Investigaciones Cardiovasculares (CNIC), 28029 Madrid, Spain; 4 Facultad de Medicina, Universidad Complutense de Madrid, 28040 Madrid, Spain; 5 IIS-Fundación Jiménez Díaz, 28040 Madrid, Spain

**Keywords:** molecular biology, gene, pulmonary arterial hypertension, pulmonary artery aneurysm, aortic aneurysm

## Abstract

Pulmonary arterial hypertension (PAH) is a rare disease caused by pulmonary vascular remodeling. Current vasodilator treatments have substantially improved patients’ survival. This improved survival has led to the appearance of complications related to conditions previously underdiagnosed or even ignored, such as pulmonary artery aneurysm (PAA). The presence of a dilated pulmonary artery has been shown to be related to an increased risk of sudden cardiac death among PAH patients. This increased risk could be associated to the development of left main coronary artery compression or pulmonary artery dissection. Nevertheless, very little is currently known about the molecular mechanisms related to PAA. Thoracic aortic aneurysm (TAA) is a well-known condition with an increased risk of sudden death caused by acute aortic dissection. TAA may be secondary to chronic exposure to classic cardiovascular risk factors. In addition, a number of genetic variants have been shown to be related to a marked risk of TAA and dissection as part of multisystemic syndromes or isolated familial TAA. The molecular pathways implied in the development of TAA have been widely studied and described. Many of these molecular pathways are involved in the pathogenesis of PAH and could be involved in PAA. This review aims to describe all these common pathways to open new research lines that could help lead to a better understanding of the pathophysiology of PAH and PAA and their clinical implications.

## 1. Introduction

Pulmonary artery (PA) dilatation is a common finding among pulmonary arterial hypertension (PAH) patients [1]. Indeed, the detection of a dilated PA often leads to the diagnosis of PAH [2]. Pulmonary artery aneurysm (PAA) is defined as a main PA dilatation above 40 mm diameter [3,4,5] and has been classically diagnosed in post-mortem studies [6]. However, in recent years its detection has increased [3]. This increasing detection is particularly prominent among PAH patients in whom PAA frequency is around 38% and is becoming more common among those patients with long-standing PAH [1]. Some studies relate PAA development to hemodynamic severity among PAH patients [5]. However, this finding is controversial since other published works have shown that a worse hemodynamic profile does not necessarily lead to PAA development [1]. Furthermore, it seems that PA pressure reduction with pulmonary vasodilators is not able to stop PA enlargement in PAH patients [2,7]. PA dilatation is related to an increased risk of sudden cardiac death [8] and among patients with PAA a non-significant higher proportion of sudden cardiac death has been described [1]. This increased risk of sudden cardiac death may be related to the presence of life-threatening complications such as PA dissection [9,10] and left main coronary artery compression [11].

Thoracic aortic aneurysm (TAA) is a well-known condition characterized by a progressive aortic enlargement, which often leads to potentially fatal complications such as aortic dissection [12]. TAA is formally defined as a dilatation of the aorta of at least a 50% in diameter compared with the normal diameter [13,14]. There are several acquired conditions leading to this abnormal growth including classical cardiovascular risk factors, vasculitis, or trauma [15]. In this regard, genetics also plays an important role. Approximately, 20% of TAA patients have a family history of the disease with 25% of them carrying a genetic variant affecting proteins involved in the extracellular matrix (ECM), vascular smooth muscle cells (SMC), or the transforming growth factor β (TGF-β) pathway. Indeed, TAA histology is characterized by the loss of SMC in the vessel media layer and the destruction of the ECM by metalloproteinases [16]. Genetic variants associated with TAA are often genetic determinants of complex syndromes affecting multiple organs and systems [17].

To our knowledge, genetic variants related to PAA have not yet been described. In fact, the identification of mutations classically related to familial PAH (*BMPR-2*, *TBX4*, *KCNK3*) are not more frequent among patients with a PAA [1]. Furthermore, although some molecular pathways altered in PAH have been shown to be somehow implicated in great vessel aneurysm development (e.g., increased ALK1 receptor pathway activation in TAA [18], *ENG* mutations in some patients with hereditary hemorrhagic telangiectasia [19] and TAA or CAV1 and AQP1 implication in abdominal aorta aneurysm [20,21]), their association with PAA has not been described. However, some of the molecular pathways related to the development of TAA have been described to have a role in PAH pathogenesis [17]. In this review we aim to summarize these common molecular pathways to help researchers identify potential molecular targets that could be involved in the development of PAA among PAH patients.

## 2. Genetic Variants in Thoracic Aortic Aneurysms

At the time of writing this review, there are 29 identified genes that have been shown to be associated with TAA development. These genes usually encode for components of the ECM, the TGF-ß pathway, or are involved in SMC function [17]. The genes affected in TAA include: *ACTA2, BGN, COL1A2, COL3A1, COL5A1, COL5A2, EFFEMP2, ELN, EMILIN1, FBN1, FBN2, FLNA, FOXE3, LOX, MAT2A, MFAP5, MYH11, MYLK, NOTCH1, PRKG1, SKI, SLC2A10, SMAD2, SMAD3, SMAD4, TGFB2, TGFB3, THFBR1,* and *TGFBR2.*

The identification of these mutations is useful not only for family screening but also for prognostic implications derived from a portion of variants and genes with well-established genotype–phenotype correlations [17]. Based on the increased risk for acute aortic syndrome, the diameter threshold, which marks the indication for surgery, can change depending on the molecular etiology [17,22].

The hereditary influence that causes or predisposes a person to systemic artery dilation can be divided among (i) those carrying a highly penetrant variant and frequently showing syndromic features (e.g., FBN and Marfan syndrome, TGF-β and Loeys–Dietz syndromes or collagen and Ehlers–Danlos syndrome (Table 1)); (ii) those with less penetrant and perhaps more frequent variants in whom environmental influence might be key to aneurysm development (e.g., rare variants in MYH11 and ACTA2 associated with familial and sporadic non-syndromic TAA); and (iii) a group of patients not carrying a net hereditary risk charge for aneurysm development who need a heavy environmental influence for aneurysm development (elderly, hypertension, atherosclerosis) [23]. These subdivisions are an adaptation to the threshold theories of genetic risk and might be applicable to PAA development. However, more research is necessary in the field for further conclusions in this regard.

## 3. Extracellular Matrix Dysregulation in the Genesis of Pulmonary Vascular Remodeling

In the last few years, the ECM has been shown to play an essential role in the pathogenesis of PAH. Changes in the ECM seem to precede the appearance of pulmonary vascular remodeling. These changes in the ECM include increased collagen deposition or cross-linkage and disruption of the elastic laminae [24]. ECM remodeling is an early phenomenon in the pathogenesis of the disease and it is suspected to be a cause of the pulmonary vascular remodeling instead of a consequence of increased afterload [24]. Thus, increased PA stiffness triggers pulmonary vascular remodeling by activating different signaling pathways [24]. The most common molecular pathways altered in PAH include the bone morphogenetic protein (BMP), TGF-β pathway, abnormal micro-RNA expression, increased growth factors (platelet-derived and vascular endothelial growth factors among others), altered inflammatory pathways (interleukins IL-6, IL-1β), mitochondrial metabolism, other neurohormonal pathways (i.e., serotonin, adrenergic), prostacyclin/cAMP/PKA, endothelin/calcium signaling, and NO/sGC/cGMP/PDE5 all leading to endothelial dysfunction and abnormal SMC proliferation and reduced apoptosis [25].

## 4. Molecular Pathways Affected in Both Pulmonary Hypertension and Aortic Thoracic Aneurysms

**Figure 1:**
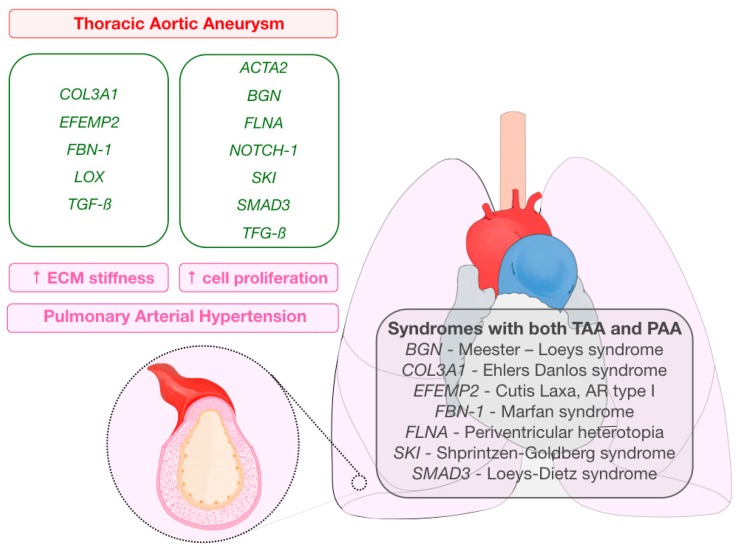
Graphical abstract representing genes related to thoracic aortic aneurysms that also encode for proteins implicated in the pathogenesis of pulmonary arterial hypertension (extracellular matrix stiffness (left column), increased cell proliferation (right column)). Syndromes in which pulmonary and aortic aneurysms may be present are also listed.

As described before, for both TAA and PAH, histological changes affecting the ECM might participate on the origin of the disease. Some of the genes that have been shown to be related to TAA development encode for proteins, which, in the absence of described mutations, are somehow involved in PAH pathogenesis (Table 1). Genes are classified depending on their potential effects on PAH development: increased ECM stiffness or increased cell proliferation (Figure 1).

## 5. Genes Potentially Related to Increased ECM Stiffness

***COL3A1:*** Mutations in collagen III chains are responsible for Ehlers–Danlos Syndrome type IV [61]. This syndrome is characterized by fragility of connective tissue with clinical manifestations affecting the skin, joints, blood vessels, and other organs [62]. In PAH there is an increased deposition of insoluble collagen in the ECM of lung vasculature with an increased expression of COL14A1, COL4A5, and COL18A1, which contributes to reduced pulmonary artery compliance in these patients [24]. However, PAH has not been described among Ehlers–Danlos syndrome patients, but further exploration of the role of collagen mutations in both PAH and PAA development might be useful for a better understanding of these conditions.

***EFEMP 2***: Fibulin-4 (encoded by the *EFEMP 2* gene) is an ECM protein responsible of stabilizing matrix structures through intramolecular bridges. Mutations in this protein cause a disruption of the ECM with shortened and reduced elastin fibers [31]. Genetic variants in the fibulin-4 gene cause a syndrome called cutis laxa AR type I. Patients with this syndrome present multiple artery aneurysms and other systemic alterations [35]. No alterations in fibulin-4 have been described in the pathogenesis of PAH. In contrast, fibulin-5, a protein necessary for an adequate elastin fiber assembly in the ECM, has been shown to be upregulated in mice with PAH [37].

***FBN-1***: Marfan syndrome is a well-known heritable disorder caused by a mutation in fibrillin-1 affecting different organs and systems, with TAA and aortic dissection the main cause of morbidity and mortality in these patients. Fibrillin-1 is a glycoprotein of the ECM where it forms microfibrils and is present in the connective tissue of different parts of the body. However, fibrillin is not only a structural component of the ECM. It plays an important signaling role with TFG-β, which contributes to the multisystemic disturbances in Marfan syndrome [63]. In fact, TGF-β plays a main role in endothelial and smooth muscle cells proliferation and in the angiogenesis process, which explains its implication in the pathogenesis of PAH [59]. Despite the alterations described in the TGF-β pathway, PAH is not a common manifestation among patients with Marfan syndrome [38].

***LOX***: Lysyl oxidase is an oxideaminase implicated in the cross-linking of elastin and collagen fibers. Patients with a mutation in this gene present early development of TAA and dissection [45]. When lysyl oxidase activity decreases, histologic findings in the aortic wall of these patients include fragmented elastic fibers and an aberrant smooth muscle layer [45]. Lysyl oxidase is also involved in the development of pulmonary vascular remodeling, but in this case, the development of these histological changes is caused by an increased expression in the lung tissue of hypoxia–exposed mice or monocrotaline-exposed rats and in PAH patients [46]. Lysyl oxidase causes an increased cross-linking of collagen in the lung vasculature [64]. Inhibition or down-regulation of lysyl oxidase attenuates these histological changes [46,64]. Histological changes in the great pulmonary vessels related to the increased activity of LOX among PAH patients have not been evaluated. However, increased LOX activity has been shown to be associated with enhanced oxidative stress leading to increased vascular stiffness and elastin alterations leading to vascular complications [47]. This increased vascular stiffness might be responsible for an abnormal adaptation to increased wall tension among PAH patients leading to PA dilatation.

***TGFBR1* and *TGFBR2***: Mutations in TGF-β pathway inherited in an autosomal dominant pattern are responsible of the development of Loeys–Dietz syndrome. This syndrome includes the development several vascular complications (aneurysms and dissections) and a variety of systemic features [41,57,65,66]. The contribution of the TGF-ß signaling pathway to the development of PAH is well-known as increased activity is a classic finding among these patients, enhancing smooth muscle cells and endothelial cells proliferation through SMAD3 and SMAD2 activation [59]. PAH has not been described among patients with Loeys–Dietz syndrome, but TGF-β pathway evaluation could help to understand the pathogenesis of PAA among these patients.

## 6. Genes Potentially Related to Increased Cell Proliferation

***ACTA2***: This isoform of the contractile protein α—actin defines the differentiation of SMCs into functional SMCs. Heterozygous missense mutations in *ACTA2* cause vascular disorders secondary to an abnormal proliferation of SMCs. These disorders include familial TAA, moyamoya syndrome, and multisystemic smooth muscle dysfunction. All of these have a dominant pattern of inheritance and are characterized by vascular manifestations including aneurysms and occlusive lesions [26,27,28]. Hyperproliferation of SMCs in the intimal or medial layers of lung vessels cause a reduction of the lumen that leads to the appearance of histological findings consistent with PAH. These changes were observed both in patients with and without previous history of patent ductus arteriosus and in some cases are related to the presence of a PAA [26,29].

***BGN***: Mutations in *BGN*, the gene encoding the protein biglycan, cause Meester–Loeys syndrome. Patients with this syndrome present early-onset aortic aneurysms and dissection associated with extravascular alterations [30]. Biglycan is a small leucine-rich proteoglycan. It’s a component of the ECM and also plays a main role in multiple signaling pathways and it’s involved in several human diseases due to its capability to interact with different growth factors and cytokines [67]. Interestingly, one of the growing factors that interacts with biglycan is the bone morphogenetic protein 2 (BMP-2), enhancing the BMP-2 signaling in osteoblastic cells [32]. Whether this mutation may influence in the development of PAH is unknown, with no cases described in the literature. However, it seems an interesting candidate to further explore regarding PAH and PAA.

***FLNA***: Filamin A is an actin-binding protein that interacts with integrins, receptors, and messengers to regulate the organization of the actin cytoskeleton, which regulates motility, adhesion, and division of the cell [68]. Mutations in *FLNA* cause a variety of syndromes affecting the cardiovascular system (periventricular nodular heterotopia [42], X-linked cardiac valvular dysplasia [43]), osteoarticular abnormalities (Melnick–Needles syndrome, oto-palato-digital syndrome, frontometaphyseal dysplasia), or gastrointestinal disturbances [68]. Regarding the respiratory system, different articles have reported the presence of lung disease presenting hyperinsufflation and emphysematous changes in the histologic evaluation but without vascular remodeling, suggesting PAH [68]. PAH is not a common finding in patients with genetic variants in *FLNA*, with only two cases described in the literature (two sisters with PAH, PAA compressing the left main coronary artery and an identified splicing mutation in the filamin A gene) [44]. Whether *FLNA* mutations are commonly related to PAH and PAA development is unknown, but given the presence of familial cases of PAH and PAA we believe this pathway should be investigated.

***NOTCH 1***: Genetic ablation of *NOTCH1* has been found to impair endocardial-to-mesenchyme transition causing cardiac alterations in mice. Furthermore, these mice present a more severe valve calcification [49]. Mutations in *NOTCH1* have been identified in familial cases of bicuspid aortic valve, TAA, and aortic dissection [48,49] and in Adams Oliver syndrome, which includes several congenital heart defects and pulmonary hypertension related to pulmonary vein stenosis [50,51]. *NOTCH1* is also involved in the pathogenesis of PAH with an increased expression in lungs of PAH patients and Sugen-hypoxia exposed rats by down regulating p21 and Bcl-2 which leads to a reduced apoptosis of endothelial cells [52].

***SKI***: Mutations in the Sloan–Kettering proto-oncogene lead to multiple disorders in different organs and systems (skeletal, neurologic, cardiovascular, and connective tissue) [54]. This proto-oncogene downregulates the TGF-ß signaling, which is implicated in the development of TAA. Thus, mutations in the *SKI* leads to aortic enlargement [54]. TGF-ß signaling pathway activation is related to increased SMC and endothelial cells proliferation leading to the development of PAH [59,69]. The presence of PAH or PAA among patients with mutations in *SKI* has not been described. It seems interesting to further explore its potential protective role in this disease considering its direct connection to the TGF-β pathway.

***SMAD3***: SMAD protein mutations lead to different syndromes associated with TAA and dissection in addition to other vascular disturbances accompanied by several extravascular complications (Loeys–Dietz syndrome 3 and juvenile polyposis/hereditary hemorrhagic telangiectasia syndrome) [55]. This family is involved in the development of pulmonary vascular remodeling acting as a downstream modulator of the TGF-β pathway. SMAD2 and SMAD3 promote fibrosis induced by TGF-β signaling causing tissue fibrosis while SMAD7 acts as a negative modulator [70]. However, in PAH patients and in animal models of PAH, a loss of SMAD3 function increased pulmonary vascular remodeling by disinhibiting the myocardin-related transcription factor [56]. Nevertheless, pulmonary hypertension has not been described among patients with SMAD3 or SMAD4 mutations.

## 7. Certain Syndromes May Present Aortic and Pulmonary Artery Aneurysms

In addition to the shared pathways between familial or syndromic TAA and PAH, some of these syndromes are related to the development of PAA in normotensive patients. Genetic variants associated with these syndromes and their clinical features are detailed in Table 1.

**Meester–Loeys syndrome**: A single case of PAA in a patient with this condition has been described, with concomitant diagnosis of mitral valve prolapse [30].

**Ehlers–Danlos syndrome**: A single case of a patient with a PAA has been reported among Ehlers–Danlos syndrome patients [34]. Furthermore, the presence of PAA seems to be less frequent among connective tissue disease-related PAH patients [1].

**Cutis laxa, AR type I**: This ECM alteration is responsible for a syndrome with different clinical features including PAA [31,36].

**Marfan syndrome**: The dilatation of the pulmonary artery was a minor cardiovascular criteria for Marfan Syndrome diagnosis until the definition of the revised Ghent nosology, where it was not included [38,71]. However, up to a 74% of patients with Marfan Syndrome present a PA dilatation [72] and 15% a PAA [73], including PAA and PA dissection, which confirms that histological changes also affect the pulmonary vasculature [39,40].

***FLNA* mutations**: As previously stated, only two cases of familial PAH with PAA have been described in the literature [44]. However, the presence of PAA among patients with filamin cardiac variants is not common.

**Shprintzen–Goldberg syndrome**: A single case of PAA has been reported among patients with mutations in the Sloan–Kettering proto-oncogene [53].

**Loeys–Dietz syndrome**: The diagnosis of PAA among Loeys–Dietz syndrome patients is not as common as it is for the Marfan syndrome patients, being limited to a single reported case [58,60]. However, in some patients, a concomitant diagnosis of TAA and PAA has been described with similar histological changes described in both arteries [74].

## 8. Hemodynamic Overload Contributes to Development of Both Aortic and Pulmonary Aneurysms

Hypertension and PAH are common causes for the development of TAA and PAA, respectively. However, not every patient exposed to an increased intravascular pressure develops a great vessel aneurysm. Furthermore, the effect of antihypertensive drugs or pulmonary vasodilators on artery diameter change is debatable [2,7]. Thus, besides increased intravascular pressure, patients who finally develop abnormal artery growth must present a genetic susceptibility triggering ECM changes responsible of aneurysm development.

Hypertension is a well-known risk factor for aortic root enlargement and TAA [75]. Cystic medial degeneration or necrosis are the typical histological findings in patients with TAA and dissection and its appearance is accelerated by aging and hypertension [76]. Moreover, a long exposure to hypertension seems to play an important role in TAA development since the prevalence among untreated patients with recent diagnosis is low [77]. Contradictory results about the influence of high blood pressure in the development of TAA have been described [78]. Nevertheless, cumulative evidence seem to support the fact that the aortic root dilatation is hypertensive-related organ damage related to a worse prognosis among patients with high blood pressure [78]. Antihypertensive drugs are commonly used to slow the aortic root enlargement among Marfan syndrome patients. Propranolol, a non-selective beta-blocker, has been shown to reduce aortic root growth and clinical events in this population [79]. Treatment with the angiotensin-II receptor blocker losartan was shown to be effective in stopping the progressive enlargement of the aortic root in preclinical mice models of Marfan syndrome [80]. However, controversial results have been obtained in different clinical trials, with no differences in effectiveness when compared to betablockers [81,82,83,84]. Whether antihypertensive drugs are effective in the reduction of aortic dilatation among the hypertensive population is still debatable [78]. However, based in the evidence obtained in clinical trials with Marfan syndrome patients, current guidelines recommend the use of betablockers as the preferred drug in hypertensive patients with aortic enlargement [85].

Physiopathological data about PAA development is scarce. PAA appears as a consequence of different diseases [86], being more common among patients with PAH especially among those with congenital heart defects [1]. In PAH, histological damage is not confined to lung capillaries. Structural changes affecting the ECM on the PA wall, with increased collagen content and an increased elastolytic activity, leads to an increased PA stiffness that affects the ventricular–arterial coupling and could be responsible of the PAA development [87]. However, despite PAA being common among PAH patients, hemodynamic severity of PAH has not shown to be an independent risk factor for PAA development in this population [1]. Furthermore, effective PA pressure reduction with pulmonary vasodilators have failed to stop progressive PA enlargement [2,7]. Thus, it seems that a chronic exposure to an increased afterload worsen irreversible ECM changes leading to PA growth [1]. Whether there is a genetic predisposition for these changes, and whether the molecular pathways affected in these patients are the same as those affected in TAA patients is still unexplored.

## 9. Diameter-Based Indication for Prophylactic Surgery: Do We Need a Variant-Dependent Approach for PAA?

In addition to losartan and beta-blockers, there are no available treatments to stop TAA enlargement and to reduce the incidence of fatal complications, especially aortic dissection. For this reason, current management is based on the identification of patients at a higher risk for complications to perform a prophylactic resection surgery [22]. The indication for this type of surgery is based both on the presence of an identified mutation and on aortic diameter or fast growth [17,22]. However, the suitability of these recommendations is widely discussed as the diameter of TAA in patients with acute dissection is lower than 55 mm in the majority of the cases and lower than 50 mm in 40% of the cases [88]. Thus, the indication of early surgery should be based on the identification of high-risk patients depending on the presence of specific mutations, the diagnosis of certain syndromes, or family history of aortic dissection.

The indication for resection surgery becomes even more difficult when talking about PAA. In this setting, the lack of strong evidence is added to the high surgical risk for PAH with up to a 25% mortality risk for cardiac surgery [89]. The risk of sudden cardiac death among PAH patients reaches 30% [90]. Although an increased PA diameter has been shown to be an independent risk factor for sudden cardiac death in PAH patients [8], there are no necropsy registries allowing for the confirmation of PA dissection being the main cause of sudden death in this population. Furthermore, there are some case series of patients with confirmed PA dissection in whom a conservative strategy was chosen with an acceptable long-term survival and who presented with some PA branches dissection without a severely dilated main PA [9,10]. Furthermore, there are other conditions that could be responsible of an unexpected death in PAH patients, such as left main coronary artery compression by a PAA, which could induce myocardial ischemia and fatal ventricular arrhythmias [11]. Some authors have proposed recommendations for prophylactic surgery in patients with a PAA, which include a diameter threshold of 55 mm or even the diagnosis of PAH [3,4]. However, in Marfan syndrome patients, the indication for elective surgery over the PA is uncommon and reserved for those patients with a PA diameter larger than 60 mm, as few complications related to PA enlargement have been described in these patients [72]. Furthermore, left main coronary artery compression, which could explain an important proportion of sudden deaths in patients with a PAA, can be properly treated with percutaneous procedures, avoiding the extremely high surgical risk [11]. Thus, the evidence available does not support a recommendation for early surgery in PAH patients based on the PA diameter. The identification of mutations related to a higher risk of PAA and its complications could be helpful in the decision-making process in PAH patients with a PAA. Tissue and molecular evaluation of the PA of patients undergoing lung transplant or autopsy studies is essential to improve our knowledge.

## 10. Future Trends for Research and Patient Care

According to this comprehensive review, PAA might be another manifestation of PAH, related to changes in molecular pathways and not only a consequence of the hemodynamic overload [24]. This hypothesis is reinforced by the fact that some of these pathways are also altered in patients with familial or syndromic TAA [17]. To summarize, changes in the ECM are responsible for an increased arterial stiffness, which may lead to both great vessels enlargement and pulmonary vascular remodeling.

The management of patients with TAA has evolved in recent years thanks to advances in clinical genetics and in the development of multidisciplinary teams specialized in this field. Several mutations have been identified that are not only related to the development of a TAA, but also to a greater frequency of aortic dissection. Despite hypertension being a classic risk factor for the development of TAAs, antihypertensive treatment has not been able to demonstrate its effectiveness to stop aneurysm growth or prevent the development of complications [78].

PAA is a growing diagnosis among patients with PAH. Until recently, it was considered a rare diagnosis [6], limited in many cases to a necropsy finding. However, in recent years, the survival of PAH patients has improved markedly [91] and PAA diagnosis has increased, with a frequency of diagnosis up to 38% among PAH patients [1]. The pathophysiological mechanisms leading to the development of a PAA are unknown. Nevertheless, there is strong evidence about the involvement of the extracellular matrix in these patients, with histological findings similar to those described in aneurysmal aortas of hypertensive patients [92]. However, as for the TAA, the optimization of pulmonary vasodilator treatment has not been able to slow the growth of the pulmonary artery. On the other hand, in the case of the PAA, we lack information about specific mutations that would help predict their development and the risk of complications. In addition, in the case of PAA in patients with PAH, decision-making is especially complex. Clinicians are forced to choose between an unknown risk of sudden death and an extremely high surgical risk.

Current treatment for PAH is based on the dilatation of pulmonary vasculature [93]. Despite good results in terms of improved survival, functional class, and quality of life, these therapies fail to cure the disease since they are not acting on the causative physiopathological mechanism. Furthermore, they are not able to stop the progressive PA enlargement in these patients, which could lead to fatal complications. For this reason, it is imperative to understand the PAH pathophysiology as a combined phenomenon in which both pulmonary vascular remodeling and ECM disturbance constitute a vicious cycle that leads to ventricle–arterial dysfunction. Based on the reviewed literature, we hypothesize that early changes in the ECM might contribute to further development of PAH. These ECM changes would include increased insoluble collagen deposition (COL14A1, COL4A5, COL18A1) and elastic fiber disruption or disassembly (fibulin 5, fibrilin, LOX, TGF-β) leading to increased PA stiffness. In later stages, increased afterload secondary to pulmonary vascular remodeling leads to the dilatation of a non-compliant PA. Additionally, the TGF-β pathway (which involves other PAH genes such as ALK1, BMP9, or Smad family members) has been widely studied in the development of aortic aneurysms [18]. With all this strong evidence in place, we speculate that the TGF-β pathway might play a relevant role, inducing angiogenic factors that contribute to ECM degradation within the PA. An alteration of this pathway might therefore contribute to damage of the vessel wall (including fibroblasts, smooth muscle, and endothelial cells), already subjected to high afterload and therefore contributing to dilation and aneurysm. In addition, it will be interesting to further explore if an aberrant SMC phenotypic switching that is able to induce excessive proliferation and metalloproteinase production that degrades the ECM and increased extracellular vesicles secretion that promotes vascular inflammation and calcification (such as in the TAA [94]), might have a role in the development of PAA. Since PAH is usually diagnosed at advanced stages, with established non-reversible tissue changes, by the time pulmonary vasodilator therapy is started, the PA wall is already damaged. For this reason, current vasodilator therapy is unable to stop progressive PA enlargement.

In this field, the evaluation of molecular pathways, widely studied in familial or syndromic TAA and that have been shown to play a role in the development of PAH, could improve our understanding of the pathophysiology of PAH and PAA. There are currently several TAA animal models based on some of the described genes (COL3A1, EFEMP2, FBN-1, LOX, BGN, FLNA, SMAD3, TGF-ß) [17,95]. These animal models have not been used to evaluate changes in the PA but they could be useful in this setting. This better knowledge of the disease could be helpful in developing new therapies focused on the early changes in the ECM and to identify patients at risk of developing a PAA or one of the described mechanical complications.

## 11. Conclusions

Current knowledge regarding PAA pathogenesis is scarce. TAA and PAH share molecular pathways responsible of changes in the ECM and vascular remodeling in both systemic and pulmonary circulation. The study of these common pathways might help to better understand the pathophysiology of PAH and its consequences, including PAA and its complications. Furthermore, it could help to identify novel molecular targets beyond pulmonary vasodilator therapies that would open new therapeutic avenues in the field.

## Figures and Tables

**Table 1:** Common molecular pathways between thoracic aneurysm and pulmonary hypertension or pulmonary aneurysm. BAV = bicuspid aortic valve; BMP = bone morphogenetic protein; ECM = extracellular matrix; OMIM = online mendelian inheritance in man; PA = pulmonary artery; PAH = pulmonary arterial hypertension; PH = pulmonary hypertension; SMC = smooth muscle cell; TAA = thoracic aortic aneurysm; TGF-β = transforming growth factor β ** PH and PA dilatation in Adams–Oliver syndrome related to pulmonary vein stenosis. ^^ Loss of SMAD3 function promotes vascular remodeling but PH has not been described in patients with pulmonary hypertension.

Gene (Locus, Inheritance)	Protein (Function)	Histological Findings	Phenotype (OMIM)	PAH	PA Dilatation	Potential Implications in PH
*ACTA 2* (10q23.31, AD)	Smooth Muscle α-actin (contractile protein)	SMC proliferation Elastic fibers fragmentation Vasa vasorum stenosis [26]	Multisystemic Smooth Muscle Dysfunction Syndrome (613834) [26]: - Aortic and cerebrovascular disease - Dilated pupils - Hypotonic bladder - Hypoperistalsis - Pulmonary hypertensionThoracic aortic aneurysm-6 (611788) [27]: - TAA and dissection - Livedo reticularis - Patent ductus arteriosus - BAVMoyamoya disease (611788) [28]: carotid stenosis	Yes [29]	Yes [26]	SMCs hyperproliferation [26]
*BGN* (Xq28, XL)	Biglycan (ECM component, signaling)	Normal collagen content Normal-appearing elastin fibers	Meester–Loeys Syndrome (300989) [30]: - Early TAA and dissection - Facial dysmorphism: hypertelorism, proptosis, frontal bossing, malar hypoplasia. - Pectus deformities - Joint hypermobility or contractures, - Skin striae - Bifid uvula - Cervical spine instability	No	Yes [30,31]	BMP2 signaling pathway altered [32]
*COL3A1* (2q23.2, AD)	Collagen 3 α1 chain (ECM component)	Reduced type III collagen in aorta Decrease and disorganization of elastic fibers	Ehlers–Danlos Syndrome type IV (130050) [33]: - Short stature - Lobeless ears, keratoconus, thin lips and nose - Mitral valve prolapse - Intracranial aneurysms - Early aortic dissection - Vessel fragility - Spontaneous bowel rupture	No	Yes [34]	ECM disruption [24]
*EFEMP2* (11q13.1, AR)	Fibulin 4 (elastic fibers assembly and formation)	Fibrointimal hyperplasia Decrease and fragmentation of elastic fibers	Cutis laxa, AR type Ib (614437) [31,35,36]: - Ascending aorta and other vascular aneurysms. - Lung emphysema - Multiple artery aneurysms - Facial dysmorphism: flattened faces, prominent forehead, hypertelorism - Pectus excavatum - Hypermobility - Multiple fractures	No	Yes [31]	Fibulin 5 up-regulated in mice with PAH [37]
*FBN-1* (15q21.1, AD)	Fibrilin-1 (ECM component, signaling)	Fragmented elastic lamellae Cystic medial necrosis Fibrosis Loss of SMC	Marfan syndrome (154700) [38]: - Increased height - Long limbs and digits - Scoliosis and lordosis - Overbite - Myopia, ectopia lentis - Mitral valve prolapse - Aortic dilatation - Striae distensae - Spinal arachnoid cysts, dural ectasia - Hyperlaxitude	No	Yes [39,40]	TGF-ß pathway altered [41]
*FLNA* (Xq28, XL)	Filamin A (Actin cytoskeleton)	Irregular collagen fibril	Periventricular nodular heterotopia (300049) [42]: - Epilepsy, normal intelligence - Patent ductus arteriosus - Aortic aneurysms - Bicuspid aortic valve - Connective tissue abnormalitiesCardiac valvular dysplasia (314400) [43]: - Multivalvular dysplasia and regurgitation - Hyperextensible skin and joint mobility	Yes [44]	Yes [44]	Not described
*LOX* (5q23.1, AD)	Lysyl oxidase (collagen and elastin cross-linking)	Reduced expression: Medial degeneration (loss of elastin fibers and smooth muscle cells) Increased expression: increased cross-linking collagen	Familial thoracic aneurysm 10 (617168) [45]: - TAA and dissection - BAV - Dural ectasia - Pectus deformity - Joint hypermobility - Skin striae	Yes [46]	No	Increased vascular stiffness [47]
*NOTCH1* (9q34.3, AD)	NOTCH1 transcription regulator	Degenerated elastic fibers of the media	Aortic valve disease 1 (109730) [48,49]: - Hereditary bicuspid aortic valve - TAA and dissectionAdams–Oliver Syndrome 5 (616028) [50,51]: - Aplasia cutis - Terminal transverse limb defects - Pulmonary vein stenosis, congenital heart defects - Cutis marmorata telangiectasia - Brain palsy	Yes ** [52]	Yes ** [51]	Reduced apoptosis of endothelial cells (p21 and Bcl-2 down-regulation) [52]
*SKI* (1p36.33-p36.32, AR)	Sloan Kettering proto-oncogene signaling in the TGF-β pathway	Cystic medial necrosis	Shprintzen–Goldberg syndrome (182212) [53,54]: - Craniosynostosis - Marfanoid habit - Facial dysmorphism: hypertelorism, downslating palpebral fissures, micrognathia - Hypotonia - Arachnodactyly, pectus deform, scoliosis, joint hypermobility	No	Yes [53]	Down-regulation of TGF-ß pathway leading to pulmonary vascular remodeling [54]
*SMAD3* (15q22.33, AD)	SMAD3	Fragmented and reduced elastic fibers Mucoid medial degeneration Medial collagen accumulation	Loeys–Dietz Syndrome 3 (613795) [55]: - Hypertelorism, bifid uvula - Mitral valve prolapse, left ventricular hypertrophy, atrial fibrillation - TAA and dissection, other arterial aneurysms - Pectus deformity - Hernias - Dural ectasia, spondylolisthesis, hip osteoarthritis - Arachnodacyly, pes planus	Yes [56] ^^	No	Loss of function related to increased pulmonary vascular remodeling via myocardin-related transcription factor [56]
*TGFBR1* (9q22.23, AD)	TGF-β receptor type 1	Fragmented and reduced elastic fibers Mucoid medial degeneration Medial collagen accumulation	Loeys–Dietz syndrome type 1 (609192) [57]: - Classic triad: arterial tortuosity and aneurysms, hypertelorism, bifid uvula or cleft palate - Craniosynostosis - Pregnancy-related complications - Immune disorders: allergies, asthma, rhinitis, eczema	No	Yes [58]	TGF-β pathway altered [59]
*TGFBR2* (3p24.1, AD)	TGF-β receptor type 2	Fragmented and reduced elastic fibers Mucoid medial degeneration Medial collagen accumulation	Loeys–Dietz syndrome type 2 (610168) [41]: - Arterial tortuosity, ascending aortic aneurysm and dissection, pulmonary artery aneurysm - Micrognathia, retrognathia, hypertelorism, blue sclerae - Bifid uvula - Pectus deformity, osteoporosis, malar hypoplasia, scoliosis, arachnodactyly - Translucent skin	No	Yes [60]	TGF-β pathway altered [59]

## Abbreviations

BMP/BMPR	bone morphogenetic protein/BMP receptor
ECM	extracellular matrix
PA	pulmonary artery
PAH	pulmonary arterial hypertension
PAA	pulmonary artery aneurysm
SMC	smooth muscle cells
TAA	thoracic aortic aneurysm
TGF-β	transforming growth factor-β
